# Feminine Gender Role Discrepancy Strain and Women’s Self-Esteem in Daily and Weekly Life: A Person x Context Perspective

**DOI:** 10.1007/s11199-022-01305-1

**Published:** 2022-06-13

**Authors:** Auguste G. Harrington, Nickola C. Overall, Jessica A. Maxwell

**Affiliations:** University of Auckland, Auckland, New Zealand

**Keywords:** Gender role strain, Feminine gender role stress, Feelings of femininity, Self-esteem, Women, Person x context

## Abstract

Social pressures to adhere to traditional feminine roles may place some women at risk of experiencing gender role discrepancy strain, when they behave, think, or feel in ways discrepant from feminine gender role expectations. The current research examines how person-level propensity to experience feminine gender-role discrepancy strain—feminine gender role stress (FGRS)—and contextual experiences of discrepancy strain—feeling less feminine in daily or weekly life—combine to undermine women’s self-esteem. After completing measures of FGRS, undergraduate women reported their feelings of femininity and self-esteem each day for 10 days (Study 1, *N* = 207, 1,881 daily records) or each week for 7 weeks (Study 2, *N* = 165, 1,127 weekly records). This repeated assessments design provided the first tests of whether within-person decreases in felt-femininity were associated with lower self-esteem, particularly for women who were higher in FGRS. Both higher FGRS and within-person decreases in daily/weekly felt-femininity were associated with lower self-esteem, but higher FGRS combined with daily/weekly decreases in felt-femininity predicted the lowest self-esteem (a person x context interaction). These results illustrate the importance of considering how person-level predispositions and contextual experiences of gender-role discrepancy strain combine to influence self-relevant outcomes for women.

**Supplementary Information:**

The online version contains supplementary material available at 10.1007/s11199-022-01305-1.

Gender roles guide and constrain what qualities and behaviors are considered feminine and masculine (Bem, 1974, 1981; Eagly & Wood, 1991). From early childhood, people are socialized to display qualities and behaviors consistent with gender roles (Bem, 1983; Bussey & Bandura, 1992; Egan & Perry, 2001; Raag & Rackliff, 1998), and learn the social consequences of not adhering to these roles (Bosson et al., 2009; Bussey & Bandura, 1992; Rudman, 1998; Rudman & Fairchild, 2004; Rudman & Glick, 2001; Rudman et al., 2012; Vandello et al., 2008). Moreover, despite continuous socialization pressures, gender role expectations are demanding, making it difficult for women and men to consistently conform to gender roles (Bosson et al., 2009; Pleck, 1981, 1995; Rudman & Fairchild, 2004). Consequently, the Gender Role Strain Paradigm (Pleck, 1981, 1995) emphasizes that the pressures traditional gender roles place on women and men, and the consequences of failing to conform to these roles, often cause people stress and strain, motivating thoughts, feelings, and behaviors that can be harmful to the self and others.

Gender role discrepancy strain is one form of gender role strain which has received particular attention in previous work, and is theorized to emerge within contexts where people behave, think, feel, or are perceived in ways that are discrepant from gender role expectations (Levant & Powell, 2017; Pleck, 1981, 1995). Person-level differences in the propensity to experience gender role discrepancy strain are commonly assessed using measures of gender role stress (Levant, 2022; Levant & Powell, 2017; Pleck, 1995). Gender role stress indexes the degree to which women and men experience stress in gender role discrepant contexts, such as when men fail to possess power or women fail to be nurturant or attractive (Eisler & Skidmore, 1987; Gillespie & Eisler, 1992). Context-level experiences of gender role discrepancy strain are most often assessed experimentally by placing women and men in gender role discrepant contexts, such as by providing gender-incongruent feedback to undermine feelings of masculinity or femininity (e.g., Berke et al., 2017; Bosson et al., 2012; Mori et al., 1987). Prior examinations have shown that both person-level and context-level gender role discrepancy strain lead to negative outcomes, such as aggression, depressed mood, and restricted eating (e.g., Bosson et al., 2012; Eisler & Skidmore, 1987; Gillespie & Eisler, 1992; Mori et al., 1987).

We propose three important gaps in this body of work that limit understanding of how experiences of gender role discrepancy strain likely shape important outcomes. First, there is a large imbalance in prior research investigating discrepancy strain processes for men compared to women. This imbalance is powerfully illustrated by a comparison of the citations of the Masculine Gender Role Stress (MGRS; 858 citations as of March, 2022) versus Feminine Gender Role Stress (FGRS; 226 citations as of March, 2022) measures. Second, person-level and context-level gender role discrepancy strain are typically examined separately, with investigations focusing on the effects of either person-level differences in gender role stress (e.g., Moore et al., 2008) or experimental manipulations of gender role discrepant contexts (e.g., Bosson et al., 2012). Yet, as we detail below, person-level propensity for experiencing discrepancy strain should predict relevant outcomes most strongly when people encounter gender role discrepant situations (a person x context interaction). Third, assessments of context-level gender role discrepancy strain have typically involved lab-based manipulations, but it is also important to examine the outcomes of feelings of gender role discrepancy strain in naturally-occurring daily or weekly contexts.

The current studies address these gaps by testing whether women higher in FGRS report lower self-esteem particularly on days (Study 1) or weeks (Study 2) they experience decreases in feelings of femininity. Given prior research has primarily focused on the outcomes of gender role discrepancy strain for men, we first describe research focused on masculine gender role stress and strain to illustrate why it is important to apply a person x context perspective. Rather than contrast, propose, or test different processes for women and men, our aim in discussing masculinity is to articulate key gender role discrepancy strain processes to illustrate the viability and utility of the approach we apply in the current studies. We then apply our person x context perspective to feminine gender role strain, and outline why we focused these initial studies on self-esteem, which is theorized to be a principal outcome of gender role discrepancy strain (Pleck, 1995). We then report two repeated sampling studies that test whether greater person-level discrepancy strain (higher FGRS) and greater context-level discrepancy strain (lower felt-femininity) combine to predict greater decreases in daily and weekly levels of self-esteem.

## A Person x Context Perspective on Masculine Gender Role Discrepancy Strain

Traditional masculine gender roles comprise the possession and demonstration of qualities related to power and status, such as agency, assertiveness, toughness, independence, and dominance (Bem, 1974, 1981; Eagly & Wood, 1991; Eagly et al., 2020; Mahalik et al., 2003; Thompson et al., 1992). Masculine gender role discrepancy strain occurs when men fail to live up to expectations of traditional masculinity, such as admitting feelings (failing to be tough), letting someone else take control (failing to be assertive and dominant), and having to ask for help (failing to be agentic and independent; Eisler & Skidmore, 1987). The MGRS scale (Eisler & Skidmore, 1987) assesses individual differences in men’s propensity to experience strain in such gender role discrepant contexts, including contexts that involve (1) physical inadequacy, (2) emotional expressiveness, (3) subordination to women, (4) intellectual inferiority, and (5) performance failure. Pleck (1995) original conception highlighted that gender role stress is an important measure of person-level differences in gender role discrepancy strain, and since then the efficacy of gender role stress measures for capturing person-level gender role discrepancy strain has been well established (Levant & Powell, 2017). Higher levels of MGRS are associated with a range of negative outcomes, such as anger, risky health behaviors, and aggression toward intimate partners (Eisler et al., 1988, 2000; Franchina et al., 2001; Moore et al., 2008).

In addition to person-level associations, the effects of context-level gender role discrepancy strain on harmful outcomes have been illustrated by placing men in gender role discrepant contexts in order to undermine their feelings of masculinity. For example, men who are asked to complete feminine tasks, told they have been outperformed by women, or received feedback they are more like women than men, exhibit more hostile cognitions and aggressive behavior compared to control conditions (Bosson et al., 2009, 2012; Cohn et al., 2009; Vandello et al., 2008). Other research has examined the effects of naturally-occurring gender role discrepant situations. For example, experiencing lower power in intimate relationships is associated with drops in men’s feelings of masculinity, which in turn predicts greater aggressive behavior towards intimate partners (Overall et al., 2016). Such aggressive responses to masculine discrepancy strain are theorized to emerge as an overt demonstration of power and thus an attempt to restore felt-masculinity.

This prior research has demonstrated that person-level and context-level gender role discrepancy strain have harmful outcomes for men in isolation. However, the person-level propensity for experiencing discrepancy strain should predict negative outcomes most strongly within gender role discrepancy contexts (a person x context interaction). Indeed, a central prediction of MGRS theory is that men higher in MGRS should be most likely to exhibit negative outcomes when they feel they are failing to live up to gender role expectations (Eisler & Skidmore, 1987). To illustrate the relevance of this approach, one study by Harrington et al. (2021) demonstrated that men higher in MGRS were more likely to report enacting aggression towards their intimate partner, but these links emerged most strongly in the gender-role discrepant context of low relationship power (relative to high power). In contrast, people lower in the propensity for gender role strain—those lower in MGRS—reported low aggression regardless of whether they faced gender-role discrepant contexts. Consistent with calls for this type of contextual application to further understanding of gender role strain (Deaux & Major, 1987; Eckes & Trautner, 2012; Levant & Powell, 2017; O’Neil, 2008; Smiler, 2004; Whorley & Addis, 2006), this rare person x context application illustrates that person-level gender role strain is most likely to predict negative outcomes in relevant gender role discrepant situations (in this case, low relationship power), and that context-level strain will predict negative outcomes most strongly for people who have a greater sensitivity to gender role strain (i.e., for those high but not low in MGRS).

## Applying a Person x Context Perspective to Feminine Gender Role Discrepancy Strain

In contrast to the breadth of findings described above for men, there is a relative dearth of research investigating gender role discrepancy strain processes for women. Yet, women face many pressures and expectations to possess feminine qualities, and experience reprisals when these expectations are not met (Bem, 1983; Bussey & Bandura, 1992; Egan & Perry, 2001; Raag & Rackliff, 1998; Rudman, 1998; Rudman & Glick, 2001). Traditional feminine gender roles comprise the possession and demonstration of qualities related to nurturance and deference, such as passivity, communality, dependence, and attractiveness (Bem, 1974, 1981; Eagly & Wood, 1991; Eagly et al., 2020; Levant et al., 2007). As no person can always embody these qualities, the potential for women to encounter gender role discrepant situations, and associated gender role discrepancy strain, is likely common. For example, women may experience gender role discrepancy strain in situations involving a disagreement with a friend (failing to be nurturant), needing to act assertively (failing to be passive), being in a bad mood when interacting with others (failing to be communal), and gaining weight (failing to be attractive; Gillespie & Eisler, 1992).

The FGRS scale (Gillespie & Eisler, 1992) assesses the degree to which women find these types of feminine gender role discrepant contexts stressful. Thus, FGRS directly assesses individual differences in women’s propensity to experience strain in gender role discrepant contexts, including (1) having unemotional relationships (e.g., “Having others believe that you are emotionally cold”), (2) being unattractive (e.g., “Being perceived by others as overweight”), (3) behaving assertively (e.g., “Having to "sell" yourself at a job interview”), (4) not being nurturant (e.g., “A very close friend stops speaking to you”), and (5) fear of victimization (e.g., “Feeling that you are being followed by someone”; Gillespie & Eisler, 1992). Although fewer studies have investigated FGRS compared to MGRS, higher levels of FGRS have been associated with negative self-relevant outcomes relevant to feminine gender role expectations of attractiveness (e.g., eating disorders and body image issues; Martz et al., 1995; Mussap, 2007), or general failure to meet self-relevant social standards, such as depressed mood (Gillespie & Eisler, 1992) and shame and guilt (Efthim et al., 2001).

Even fewer studies have directly examined the effects of women’s experiences of context-level feminine gender role discrepancy strain, and these studies have produced inconsistent results. Women receiving feedback that they are more masculine or more like men (vs. a control condition) eat less in a social context, thereby presenting a desired feminine ideal (Mori et al., 1987), and express more support for victims of sexual assault, thereby identifying more with feminine social identities (Munsch & Willer, 2012). However, other studies have found null effects. For example, prior studies manipulating gender role discrepancy strain via gender identity incongruent feedback (vs. a control condition) found that women did not endorse stereotypical gender roles more strongly or did not report experiencing greater anger, shame, or guilt (Kosakowska-Berezecka et al., 2016; Vescio et al., 2021).

One reason for these inconsistent effects may be because the effect of gender role discrepant situations varies according to person-level differences in the propensity to experience discrepancy strain. Consistent with FGRS theory (Gillespie & Eisler, 1992), women’s person-level propensity for experiencing discrepancy strain (e.g., greater FGRS) should predict negative outcomes most strongly within gender role discrepant contexts (a person x context interaction). Thus, context-level gender discrepancy strain should have stronger effects for women higher in FGRS, and may have null effects for women lower in FGRS. The aim of the current studies was to apply a person x context perspective to test whether person-level discrepancy strain (FGRS) and context-level discrepancy strain interacted to predict self-relevant negative outcomes. However, rather than focusing on a single, narrow experience in the lab, we directly examined context-level discrepancy strain by assessing drops in women’s felt-femininity and self-esteem within the ecologically valid context of women’s daily and weekly lives.

Directly assessing feelings of femininity captures the core ingredient of gender role discrepancy strain—decreases in feelings of femininity. Indeed, it is possible that the inconsistent effects of lab-based studies may be because manipulations designed to reduce women’s felt-femininity, such as telling women they are more masculine or like men (Kosakowska-Berezecka et al., 2016; Mori et al., 1987; Munsch & Willer, 2012), did not appreciably lower felt-femininity. Despite the aim of these manipulations, prior studies have not checked whether this feedback actually results in women feeling less feminine. Examining varying levels of felt-femininity across days and weeks also has the advantage of capturing strain across a range of common situations that could result in women feeling less vs. more feminine, including any context in which women think, feel, behave, or are perceived in ways that are discrepant from expectations to be nurturant, communal, passive, and attractive. Moreover, given that women likely differ in their investment in different facets of femininity (Witt & Wood, 2010; Wood & Eagly, 2009), relative levels of strain and thus felt-femininity may vary across different situations for different women (Pleck, 1995). Some women may experience stronger drops in felt-femininity when they feel unattractive, and others may experience stronger drops when they are unsupportive and thus fail to be nurturant. Accordingly, in providing the first test of naturally occurring context-level gender discrepancy strain in everyday life, we assessed variations in women’s felt-femininity across days and weeks to directly assess a key marker of gender role strain—within-person drops in feelings of femininity—that could emerge across various situations and contexts.

Similarly, we directly assessed a central outcome of gender role discrepancy strain. Pleck (1995) original theorizing of the outcomes of gender role discrepancy strain emphasized self-esteem: in particular, failing to adhere to internalized pressures and expectations associated with traditional gender roles should undermine feelings of self-worth. Pleck’s theorizing fits with research highlighting how people’s self-esteem decreases in response to feedback that they have failed to adhere to valued social standards (Leary et al., 2003). Self-esteem also may be a particularly relevant outcome of feminine gender role strain because the pressures women face to be nurturant, passive, communal, and dependent, may motivate women to exhibit internalized self-relevant negative reactions (Bussey & Bandura, 1992; Rudman, 1998). By contrast, the null effects shown in experimental manipulations of context-level feminine gender role strain have often focused on more externalized reactions, such as anger and stereotype endorsement (e.g., Kosakowska-Berezecka et al., 2016; Munsch & Willer, 2012; Vescio et al., 2021). Although no prior research has tested the links between FGRS, felt-femininity, and self-esteem, some findings support our perspective that person-level (FGRS) and context-level (felt-femininity) gender role strain will combine to predict women’s self-esteem. For example, women who view themselves as more communal (an important facet of traditional femininity) experience lower daily self-esteem when they fail to behave communally (i.e., are less attentive to their partner’s mood changes; Witt & Wood, 2010). In the current studies, we provide the first tests of whether between-person differences in FGRS (person-level discrepancy strain) and daily or weekly variation in felt-femininity (context-level discrepancy strain) combine to predict women’s self-esteem.

## Current Research

The current studies address three important gaps in the gender role discrepancy literature by (1) focusing on feminine gender role discrepancy strain rather than the more oft-studied masculine gender role discrepancy strain, (2) examining how person-level and context-level discrepancy strain interact to predict important outcomes, and (3) expanding a focus on experimental manipulations of context-level discrepancy strain to the experience and outcomes of strain in daily and weekly life. The current studies addressed these gaps by providing the first tests of the links between women’s FGRS, decreases in felt-femininity, and self-esteem in their daily (Study 1) and weekly life (Study 2). After completing measures of FGRS, undergraduate women reported their feelings of femininity and self-esteem each day for 10 days (Study 1) or each week for 7 weeks (Study 2). Gathering repeated assessments of felt-femininity and self-esteem provided the means to test whether within-person decreases in felt-femininity on a given day (Study 1) or week (Study 2) was associated with lower self-esteem, particularly for women who were higher in FGRS. Applying our person x context perspective, we expected that greater person-level discrepancy strain (higher FGRS) and greater context-level discrepancy strain (lower felt-femininity) would combine to determine daily and weekly levels of self-esteem. In particular, both higher FGRS and within-person drops in felt-femininity on a given day or week should predict lower self-esteem (main effects of person-level and context-level strain), but higher FGRS combined with lower felt-femininity should predict the lowest self-esteem (person x context interaction).

In both studies, we also conducted additional analyses to illustrate that the expected effects reflected distinct processes related to femininity and feminine discrepancy strain by examining MGRS as an alternative predictor. Although the MGRS scale has not been validated for use in samples of women (Eisler & Skidmore, 1987), and may not have equivalent meaning for women, the MGRS involves common situations which many people (women and men) could find stressful (e.g., “Working with people who are brighter than yourself”, “Getting passed over for a promotion”, “Having your lover say that she/he is not satisfied”). As both FGRS and MGRS assess person-level propensity to experience stress in challenging situations, showing that the effects of FGRS are independent of MGRS provides a direct illustration that the effects are specific to the stress associated with situations involving feminine gender role discrepancy strain rather than emerging from a general tendency to find challenging situations of all types stressful. Although women who find the contexts assessed with the MGRS more stressful may also experience lower self-esteem, the link between MGRS and lower self-esteem should not be greater when women experience context-level feminine discrepancy strain. Rather, only FGRS (and not MGRS) should interact with variations in daily/weekly felt-femininity to predict daily/weekly self-esteem.

## Study 1

Study 1 focused on the daily within-person associations between women’s feelings of femininity and self-esteem. Participants completed a questionnaire assessing FGRS and MGRS, then reported on their feelings of femininity and self-esteem at the end of each day for 10 days. We expected that both higher FGRS (person-level discrepancy strain) and days involving within-person decreases in felt-femininity (context-level discrepancy strain) would be associated with lower self-esteem, but higher FGRS combined with decreases felt-femininity would predict the lowest self-esteem (a person x context interaction).

## Method

### Participants

Two-hundred and seven women enrolled in a third-year undergraduate psychology course at a large city-based university participated for fulfillment of a research requirement. Participants were told that the study explores how people think, feel, and behave in their daily life. Participants ranged from 17 to 48 years of age (*M* = 22.34, *SD* = 4.66). The self-reported ethnicity of our participants was as follows: New Zealand (NZ) European 38.0% (*n* = 79), NZ Māori 3.9% (*n* = 8), Asian 31.6% (*n* = 66), Indian 8.9% (*n* = 18), non-NZ European 5.9% (*n* = 12), Pacific Nations 3.9% (*n* = 8), Middle Eastern 2.6% (*n* = 5), and ‘Other’ 5.3% (*n* = 11). Approximately half of the participants were single (51.5%, *n* = 107), the remainder were in romantic relationships either dating (32.9%, *n* = 68), cohabiting (11.0%, *n* = 22), or married (4.6%, *n* = 10). We did not collect participants’ sexual orientation in Study 1. We aimed to recruit a large sample of women who completed the daily sampling procedure adequately by running the study for two consecutive academic years: 2019 and early 2020. Responses collected in early 2020 occurred immediately prior to the emergence of COVID-19 in the community, and before the country went into a nationwide lockdown. Estimates of sensitivity using intensive longitudinal methods (Bolger & Laurenceau, 2013) indicate that the final sample of 207 participants assessed at 10 time points provides adequate statistical power to detect small effects (*r* = .10).

### Procedure and Measures

Approval was obtained from the University of Auckland Human Participants Ethics Committee (ref no. 022559) Human Participant Ethics Committee prior to the start of data collection. Our analyses were not pre-registered. In an initial in-person session, participants were provided detailed information about the study, gave informed consent, completed scales assessing FGRS and MGRS, and were given detailed instructions for completing a web-based daily sampling procedure for the following 10 days.

#### Feminine Gender Role Stress (FGRS)

The FGRS scale was developed by Gillespie and Eisler (1992) to assess how stressful people find feminine gender role discrepant situations across five situations: having unemotional relationships (e.g., “Being considered promiscuous”), physical unattractiveness (e.g., “Finding out that you have gained 10 pounds”), behaving assertively (e.g., “Supervising older and more experienced employees at work”), failing to be nurturant (e.g., “Returning to work soon after your child is born”), and fear of victimization (e.g., “Hearing a strange noise while you are home alone”). Participants rated each item according to how stressful they would find each situation to be if they were in that situation (1 = *not at all stressful*, 7 = *extremely stressful*). Although the FGRS scale items assess five categories of feminine discrepant situations, the scale was designed to be used as an overall score to assess how stressful people find feminine gender role discrepant situations (Gillespie & Eisler, 1992). The original 39-item FGRS scale has established internal consistency (αs = .73 to .83) and test–retest reliability (*r* = .82; Gillespie & Eisler, 1992). We assessed 24 of the original 39 items to maximize attentive responding given the data collection paradigm and align with abbreviated measures now used to assess MGRS (see description below). Our primary approach was to select 5 items from each of the 5 subscales. Items removed were those that (1) were very similar to other high-loading items from the original scale development (Gillespie & Eisler, 1992), (2) involved situations that are not widely generalizable (e.g., “Being unusually tall”), or (3) we judged were likely to be very stressful for everyone and thus may not as sensitively assess feminine gender role discrepancy strain (e.g., “Hearing that a dangerous criminal has escaped nearby”). (See the Online Supplement for details on the 24 items retained, and the 15 items removed for these studies). As in prior use of the FGRS scale, items were averaged to create an overall score of FGRS, with higher scores representing greater FGRS. Internal reliability was comparable to the original paper outlining development of the full scale (*α* = .81).

#### Masculine Gender Role Stress (MGRS)

We assessed MGRS to distinguish the effects of FGRS from a general tendency to find challenging situations (not directly related to femininity) stressful. Participants completed the Abbreviated Masculine Gender Role Stress Scale (Swartout et al., 2015), which includes 15 items assessing how stressful people find masculine gender role discrepant situations across five situations: physical inadequacy (e.g., “Not being able to find a sexual partner”), emotional inexpressiveness (e.g., “Admitting that you are afraid of something”), subordination to women (e.g., “Being outperformed at work by a woman”), intellectual inferiority (e.g., “Working with people who are brighter than yourself”), and performance failure (e.g., “Finding you lack the occupational skills to succeed”). Items were averaged to create an overall score of MGRS, with higher scores representing greater MGRS. The abbreviated MGRS scale is commonly used and has established reliability and validity (McDermott et al., 2017; Swartout et al., 2015).

### Daily Measures

Participants were instructed to complete an online questionnaire at the end of each day for 10 days. Participants first reported the date of each entry, which was checked against the software-logged date and time to assess compliance. Before variable construction and data analyses, individual daily records were excluded if they had been completed too early to reflect experiences across the entire day (before 4 pm) or were completed in less than the pre-specified time necessary to accurately discriminate across variables (under 3 min). To be included in the sample, participants had to have completed 5 or more usable daily records. The 207 women who met these criteria completed on average 9.09 daily records, resulting in 1,881 daily records for analyses. The multi-level analysis used to assess daily associations between felt-femininity and self-esteem accounts for the small differences in numbers of entries across participants by weighting the final sample estimates based on the reliability of each participant’s data (i.e., participants with more daily records contribute more to the final estimates; Bolger & Laurenceau, 2013). Each daily record assessed participants’ feelings of femininity and self-esteem that day.

#### Daily Feelings of Femininity

To assess daily feelings of femininity, each day participants rated the extent to which they agreed with the statement “I felt feminine” (1 = *not at all*, 4 = *somewhat*, 7 = *very much*). As outlined in the Introduction, we measured and modelled decreases in women’s feelings of femininity in order to directly assess the experience of gender role discrepancy strain that may arise across various, idiosyncratic contexts encountered across women’s daily life. This face-valid assessment of felt-femininity is similar to prior assessments of masculine discrepancy strain during daily life (drops in felt-masculinity), which revealed the same links with aggressive behavior as those shown from experimental manipulations of context-level masculine discrepancy strain (i.e., threats to masculinity; Overall et al., 2016). Moreover, single-item assessments are common in daily sampling studies to reduce participant burden, and are appropriate, and even preferable, when the construct being measured is specific and unambiguous (see Allen et al., 2022).

#### Daily Self-Esteem

Three items adapted from the Rosenberg (1965) Self-Esteem Scale, and similar to prior daily assessments (e.g., Murray et al., 2003), measured daily levels of self-esteem. Participants rated the extent to which they agreed with the following statements that day (1 = *not at all*, 7 = *very much*): “I felt worthless” (reverse coded), “I felt like I was a failure” (reverse coded), “I felt worthwhile”. Items were averaged such that higher scores indicate greater self-esteem. The three items were internally consistent across people (see Table 1) and showed good reliability to assess change across days (Rc = .774).

**Table 1:** Descriptive Statistics, Reliabilities, and Correlations Across Measures: Studies 1 and 2

**Variables**	**Descriptive Statistics**			**Correlations**		
	*Mean*	*SD*	α	1	2	3
**Study 1**						
1. Feminine Gender Role Stress	5.138	.845	.894	-		
2. Daily Feelings of Femininity	4.348	1.003	—	.105^**^	-	
3. Daily Self-Esteem	5.012	.927	.8079	–.164^***^	.269^***^	-
4. Masculine Gender Role Stress	2.992	.848	.815	.476^***^	–.147^***^	-.176^***^
**Study 2**						
1. Feminine Gender Role Stress	5.302	.686	.810	-		
2. Weekly Feelings of Femininity	5.082	1.167	—	–.012	-	
3. Weekly Self-Esteem	5.061	1.264	.909	–.316^***^	.299^***^	-
4. Masculine Gender Role Stress	4.019	.665	.814	.608^***^	–.013	-.396^***^

Descriptive statistics for daily and weekly feelings of femininity and self-esteem are based on averages of within-person aggregates across the sampling period, and thus associated correlations represent associations with participants’ average across-day or across-week levels of felt-femininity and self-esteem. All measures were assessed on a 1–7 scale. Alpha values for daily and weekly feelings of self-esteem are based on averages of within-person aggregates across the sampling period (see text for within-person reliability)

^**^Correlations are significant at *p* < .01; ^***^Correlations are significant at *p* < .001

## Results

We conducted multilevel analyses to test the effects of FGRS (person-level discrepancy strain), the within-person associations of felt-femininity (context-level discrepancy strain), and the interaction between FGRS and felt-femininity (person x context discrepancy strain) on self-esteem. Multilevel modelling is necessary to account for the fact that entries from the same person are likely to be more correlated than entries from different people. The intraclass correlation for our main model was ρ = .43, *p* < .001; thus, 43% of the variance in self-esteem can be attributable to differences between people, leaving sufficient variability at both the between- and within-person level to warrant the use of multi-level analyses (e.g., Merlo et al., 2005). Using the MIXED procedure in SPSS 26.0, we followed the procedures and syntax outlined by Bolger and Laurenceau (2013) to account for the dependence arising from participants providing repeated measurements across the 10 days. As detailed by the annotated syntax in the Online Supplement, these models treat each daily assessment as repeated measures within each participant and specify an autoregressive error structure (AR1) to account for the within-person associations across each daily report of the dependent variable (see Bolger & Laurenceau, 2013 for further details).

Our primary analyses modeled the degree to which participants’ daily levels of self-esteem varied as a function of (a) feelings of femininity that day (person-centered), (b) FGRS (grand-mean centered), and (c) the interaction between daily felt-femininity and FGRS. All predictors were simultaneously modelled (i.e., all predictors were entered in one model). The repeated assessments of felt-femininity were person-centered by subtracting each participant’s mean level of felt-femininity across days from each daily report of femininity. By person-centering, the effect of felt-femininity represents daily variations in feelings of femininity from each person’s typical levels, and thus tests whether within-person changes in daily felt-femininity predict within-person changes in self-esteem (person-centering is standard practice in multi-level modelling; see Bolger & Laurenceau, 2013). We expected that both within-person decreases in daily femininity (context-level discrepancy strain) and higher FGRS (person-level discrepancy strain) would be associated with lower self-esteem, but that daily decreases in femininity and higher FGRS would interact to predict the lowest daily self-esteem.

**Fig. 1:**
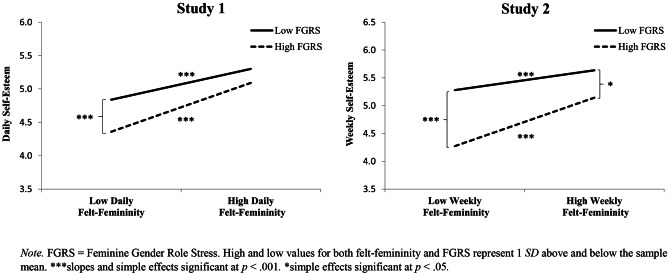
The Person x Context Interaction Between FGRS and Women’s Daily (Study 1) and Weekly (Study 2) Feelings of Femininity Predicting Self-Esteem

As shown in Table 2, within-person decreases in daily feelings of femininity were associated with women reporting lower self-esteem. Women higher in FGRS also reported lower self-esteem across days. Moreover, the significant daily felt-femininity x FGRS interaction illustrated that the within-person links between felt-femininity and self-esteem were greater for women higher in FGRS. Figure 1 (left side) displays this predicted person x context interaction. We calculated the simple effects of both person-level (FGRS) and context-level (felt-femininity) discrepancy strain by calculating the effects of low (-1 *SD*) versus high (+ 1 *SD*) levels of each variable. Focusing on the context-level effects of drops in femininity across women low versus high in FGRS, women experienced lower self-esteem on days they felt lower femininity, but this association was strongest for women higher in FGRS (dashed line: *B* = .279, *t* = 8.745, 95% CI [.216, .342], *p* < .001) compared to women lower in FGRS (solid line: *B* = .176, *t* = 6.731, 95% CI [.125, .227], *p* < .001). Focusing on the person-level effects of FGRS across days of low versus high femininity, women higher in FGRS only experienced lower self-esteem than women lower in FGRS on days they reported low felt femininity (left side of figure: *B* = –.282, *t* = –3.617, 95% CI [–.436, –.128], *p* < .001), but did not have lower self-esteem on days their felt-femininity was high (right side of figure: *B* = –.123, *t* = –1.579, 95% CI [–.277, .031], *p* = .116).

**Table 2:** Feminine Gender Role Stress and Feelings of Femininity Predicting Self-Esteem: Studies 1 and 2

**Variables**	*B*	95% CI				
		Lower	Upper	*t*	*p*	*r*
**Study 1**						
Feminine Gender Role Stress	–.177	–.326	–.027	–2.333	.021	.161
Daily Feelings of Femininity	.207	.166	.249	9.828	.000	.240
Feminine Gender Role Stress X Daily Feelings of Femininity	.061	.012	.110	2.445	.015	.062
**Study 2**						
Feminine Gender Role Stress	–.578	–.848	–.309	–4.239	.000	.315
Weekly Feelings of Femininity	.210	.152	.268	7.103	.000	.230
Feminine Gender Role Stress X Weekly Feelings of Femininity	.114	.024	.204	2.480	.013	.082

Effect sizes (*r*) were computed using Rosnow and Rosenthal (2008) formula: *r* = √(*t* 2 / *t* 2 + *df)*. In these multilevel models, the Satterthwaite approximation is applied to provide specific degrees of freedom for each effect representing the weighted average of the between and within-person degrees of freedom, which were used to calculate the effect sizes. The significant 2-way interactions between daily and weekly feelings of femininity and feminine gender role stress are shown in Fig. 1

*CI* confidence interval

### MGRS

Our second analysis tested whether the interaction between within-person decreases in femininity and FGRS was unique and independent of MGRS. Rerunning the primary analyses adding MGRS as a simultaneous predictor and moderator (see Table 3) revealed that there was no main effect of MGRS and no interaction effect between MGRS and daily femininity on self-esteem as there was for FGRS. Moreover, the interaction effect between daily feelings of femininity and FGRS predicting self-esteem shown in the left side of Fig. 1 remained significant. These results illustrate that the person x context interaction shown in Fig. 1 did not arise because women higher in FGRS were generally more likely to find any challenging situation stressful, but rather because they found specific feminine gender role discrepant situations more stressful.

**Table 3:** Feminine Gender Role Stress and Feelings of Femininity Predicting Self-Esteem Controlling for Masculine Gender Role Stress: Studies 1 and 2

**Variables**	*B*	95% CI				
		Lower	Upper	*t*	*p*	*r*
**Study 1**						
Feminine Gender Role Stress	–.112	–.281	.057	–1.308	.192	.091
Daily Feelings of Femininity	.207	.165	.249	9.725	.000	.238
Masculine Gender Role Stress	–.135	–.303	.033	–1.586	.114	.110
Feminine Gender Role Stress X Daily Feelings of Femininity	.058	.003	.113	2.070	.039	.052
Masculine Gender Role Stress X Daily Feelings of Femininity	.007	–.050	.063	.234	.815	.006
**Study 2**						
Feminine Gender Role Stress	–.220	–.548	.108	–1.327	.186	.104
Weekly Feelings of Femininity	.210	.152	.268	7.101	.000	.230
Masculine Gender Role Stress	–.608	–.945	–.270	–3.551	.001	.269
Feminine Gender Role Stress X Weekly Feelings of Femininity	.128	.020	.236	2.328	.020	.077
Masculine Gender Role Stress X Weekly Feelings of Femininity	–.026	–.134	.083	–.468	.640	.016

Effect sizes (*r*) were computed using Rosnow and Rosenthal (2008) formula: *r* = √(*t* 2 / *t* 2 + *df*). In these multilevel models, the Satterthwaite approximation is applied to provide specific degrees of freedom for each effect representing the weighted average of the between and within-person degrees of freedom, which were used to calculate the effect sizes

*CI =* confidence interval

## Study 2

Study 2 was designed to replicate Study 1 but, rather than employing daily assessments, we examined whether weekly drops in felt-femininity associated with lower weekly self-esteem, particularly for women higher in FGRS. Participants completed an initial questionnaire assessing FGRS and MGRS then reported on their feelings of femininity and self-esteem at the end of each week for 7 weeks. Again, we expected that both higher FGRS (person-level strain) and weeks involving lower felt-femininity (context-level strain) would predict lower self-esteem, but higher FGRS combined with lower weekly felt-femininity would predict the lowest self-esteem (a person x context interaction).

## Method

### Participants

One-hundred sixty-five women enrolled in a second-year undergraduate psychology course at a large city-based university elected to participate in the study (from amongst a range of other study options) for fulfillment of a research requirement. Participants were told that the study explores people’s thoughts, feelings and behavior within social interactions across their weekly life. Participants ranged from 17 to 45 years of age (*M* = 20.81, *SD* = 3.95). The self-reported sexual orientation of our participants was as follows: Heterosexual/Straight 84.2% (*n* = 139), Bisexual 10.3% (*n* = 17), Asexual 1.8% (*n* = 3), Pansexual 1.2% (*n* = 2), Gay or Lesbian .6% (*n* = 1), prefer not to say or ‘other’ 1.8% (*n* = 3). Approximately half of the participants were single (44.4%, *n* = 73), with the remainder involved in romantic relationships either dating (45.5%, *n* = 75), cohabiting (6.6%, *n* = 11), or married (3.5%, *n* = 6). We aimed to recruit as large a sample as possible to match the sample size of Study 1 by running the current study for three academic semesters. Two semesters occurred in 2019 prior to the emergence of the COVID-19 pandemic, and the third occurred after COVID-19 had initially been eliminated in the community in 2020. However, 80 participants sampled in 2020 experienced a short lockdown (18 days) during the data collection period. We included all data for transparency and to maximize statistical power, and because we did not have firm a priori expectations that the post-COVID semester would have weaker (minimize feminine discrepancy strain) or stronger (amplify strain) effects. The main and interaction effects of felt-femininity and FGRS did not significantly differ across data collected in 2019 versus 2020 (For more detailed information, see the Online Supplement). Estimates of sensitivity using intensive longitudinal methods (Bolger & Laurenceau, 2013) suggest that 165 participants assessed at 7 time points provides adequate statistical power to detect small effects (*r* = .10).

### Procedure and Measures

Approval was obtained from the University of Auckland Human Participants Ethics Committee (ref no. 022559) Human Participant Ethics Committee prior to the start of data collection. Our analyses were not pre-registered. The study was advertised to students enrolled in two large second-year undergraduate courses, which involved the possibility of participating for course credit. Students were presented with a range of studies to complete each semester, and thus this study was one of many that students could select. After signing up, participants were provided detailed information about the study and gave informed consent. Participants then completed scales assessing FGRS and MGRS and were given instructions for completing a web-based weekly sampling procedure for the following 7 weeks.

#### FGRS

The same scale used in Study 1 assessed FGRS and produced comparable descriptive statistics and reliabilities (see Table 1).

#### MGRS

In Study 2, participants completed a more detailed assessment of MGRS than the abbreviated MGRS scale used in Study 1 which consisted of 30 of the original 40 items (Eisler & Skidmore, 1987). This 30-item assessment demonstrated identical reliability to the assessment used in Study 1 (*α* = .814). Prior use of this scale in multiple studies has revealed similar means, standard deviations, and internal reliability to previous MGRS assessments (Harrington et al., 2021). See the Online Supplement for more detail on the foundation for this assessment.

### Weekly Measures

At the end of each week for 7 weeks, participants received an e-mail with a link to a questionnaire they were asked to complete as soon as possible (and preferably within 1–2 days). To remove any variation in assessment arising from participants reporting at different points during the week, the first weekly questionnaire was sent on the Friday of the week participants signed up for the study, which was, on average, 4 days (*SD* = 2.50) after completing the initial questionnaires (FGRS and MGRS). We selected Friday because it tends to represent the end of the week for most and, thus, a time that people would be able to easily reflect across their experiences that week (see Chang et al., 2018 for similar procedures).

Participants first reported the date of each entry, which was checked against the software-logged date and time to assess compliance. Before variable construction and data analyses, individual weekly entries were excluded if they were completed in less than the pre-specified time necessary to accurately discriminate across variables (under 3 min). To be included in the sample, participants had to have completed at least 5 usable weekly entries. To ensure that duplicate responses within a single week were not included, the days between each response were calculated and responses that occurred 3 or fewer days apart (and thus occurred during the same week) were deleted. For consistency, when duplicate responses were identified, the second response was deleted, and the first response was retained. These criteria and exclusions resulted in a sample of 165 women who completed on average 6.83 weekly entries, providing 1,127 weekly records for analyses. In the final sample, 122 (74.4%) completed all seven weekly questionnaires, 35 (21.3%) completed six weekly questionnaires, and 7 (4.3%) completed five weekly questionnaires. The average days between questionnaires was 7.11 days (*SD* = 1.80). Controlling for days between weekly reports did not alter any of the results.

#### Weekly Feelings of Femininity and Self-Esteem

Identical measures used to assess feelings of femininity and self-esteem in Study 1 were used in Study 2 to assess feelings of femininity and self-esteem across the past week. These measures produced comparable descriptive statistics and reliabilities as the daily assessments in Study 1 (see Table 1).

## Results

The analytical procedure of Study 2 was identical to that of Study 1. The intraclass correlation for our main model was ρ = .29, *p* < .001; only 29% of the variance in self-esteem reflected differences between people, necessitating multilevel modeling to assess within-person variation in weekly levels of self-esteem. Using the MIXED procedure in SPSS 26, we modeled the degree to which participants’ weekly self-esteem was a function of (a) feelings of femininity that week (person-centered), (b) FGRS (grand-mean centered), and (c) the interaction between felt-femininity and FGRS. All predictor variables were entered simultaneously. We expected that both within-person decreases in weekly femininity and higher FGRS would be associated with lower self-esteem, but that weekly decreases in femininity and higher FGRS would interact to predict the lowest daily self-esteem.

As shown in Table 2, within-person decreases in weekly feelings of femininity were associated with women reporting lower self-esteem. Women higher in FGRS also reported lower self-esteem across weeks. Moreover, the significant weekly feelings of femininity x FGRS interaction illustrated that the within-person links between felt-femininity and self-esteem were greater for women higher in FGRS. Figure 1 (right side) displays this predicted person x context interaction. We calculated the simple effects of both person-level (FGRS) and context-level (felt-femininity) discrepancy strain by calculating the effects of low (-1 *SD*) versus high (+ 1 *SD*) levels of each variable. Focusing on the context-level effects of drops in femininity across women low versus high in FGRS, women experience lower self-esteem on weeks they felt lower femininity, but this association was strongest for women higher in FGRS (dashed line: *B* = .301, *t* = 6.071, 95% CI [.202, .400], *p* < .001) compared to women lower in FGRS (solid line: *B* = .123, *t* = 3.734, 95% CI [.058, .188], *p* < .001). Focusing on the person-level effects of FGRS across weeks of low versus high femininity, women higher in FGRS experienced lower self-esteem than women lower in FGRS, but this effect was more pronounced on weeks they reported low feelings of femininity (left side of figure: *B* = –.735, *t* = –4.946, 95% CI [–1.029, –.441], *p* < .001), relative to weeks their felt-femininity was high (right side of figure: *B* = –.359, *t* = –2.436, 95% CI [–.649, –.069], *p* = .016).

### MGRS

Rerunning the primary analyses adding MGRS as a simultaneous predictor and moderator (see Table 3) revealed that, unlike Study 1, higher levels of MGRS were associated with lower self-esteem (a significant main effect of MGRS). Nonetheless, and critically, there was no significant interaction between MGRS and weekly felt-femininity and the FGRS and weekly felt-femininity interaction remained significant. As in Study 1, these results show that the person x context interaction shown in Fig. 1 did not arise because women higher in FGRS were generally more likely to find any challenging situation stressful, but rather because they found specific feminine gender role discrepant situations more stressful.

## General Discussion

The present studies provide the first demonstration that person-level gender role discrepancy strain—higher FGRS—and context-level gender role discrepancy strain—lower felt-femininity—combine to predict lower self-esteem in women’s daily and weekly life. In Study 1, we assessed the associations between women’s FGRS, within-person variation in felt-femininity, and self-esteem across 10 days. In Study 2, we assessed the associations between women’s FGRS, within-person variation in felt-femininity, and self-esteem across 7 weeks. As expected, in both studies, higher FGRS (person-level discrepancy strain) and within-person decreases in daily and weekly femininity (context-level discrepancy strain) predicted lower self-esteem, but higher FGRS combined with daily decreases in femininity predicted the lowest self-esteem (person x context interaction).

The results address three important gaps. First, the results emphasize that feminine gender role discrepancy strain, which is comparatively understudied compared to masculine gender role discrepancy strain, has negative implications for women’s self-evaluations. Second, by applying a person x context perspective, the results emphasize that understanding of gender-role discrepancy strain is enhanced when both person-level differences and context-level effects are examined in combination. Prior research has shown that person-level differences in the propensity to experience feminine gender role strain (e.g., FGRS) are associated with outcomes related to internalized feelings of low self-worth (e.g., greater depressed mood, shame, and guilt; Efthim et al., 2001; Gillespie & Eisler, 1992), but the current results show these person-level effects occurred most strongly in contexts when women experience gender role discrepancy strain as indicated by daily and weekly decreases in feelings of femininity. Finally, rather than focusing on a single, narrow experience in the lab, the current studies illustrated the importance of drops in feelings of femininity within the ecologically valid context of women’s daily and weekly lives. In the following sections, we elaborate how the current research expands understanding of gender role discrepancy strain processes and advances prior research.

### The Importance of Gender Role Discrepancy Strain for Women

Women face social pressures to conform to the expectations of traditional feminine gender roles. Yet, no person can always embody these strict expectations, and women are likely to encounter gender role discrepant situations in their daily and weekly lives, placing them at risk for gender role discrepancy strain and associated negative outcomes. Despite the relevance of feminine gender role strain processes to women’s wellbeing, prior research has primarily focused on the outcomes of men’s gender role discrepancy strain and relatively little research has investigated the outcomes of gender role discrepancy strain processes for women. The current research highlights the importance of this gap by demonstrating that person-level (FGRS) and context-level (drops in felt-femininity) gender role discrepancy strain combine to predict lower self-esteem in women’s daily and weekly lives.

The current research provides the first test of naturally occurring context-level feminine gender role discrepancy strain that emerges in women’s daily and weekly lives. To do so, we assess drops in feeling of femininity, which provides a specific, unambiguous measure of daily and weekly experiences of feminine discrepancy strain that could emerge from a broad array of contexts. However, future research should expand on this initial demonstration by identifying the range of specific situations and experiences that can lead to feminine gender role discrepancy strain and lower felt-femininity. Indeed, many routine situations across women’s personal and occupational lives are likely to promote feminine gender role discrepancy strain by making salient the degree to which women are embodying (or failing to embody) feminine qualities of nurturance, communality, attractiveness, passivity, and dependence. Developing assessment tools to examine the array of routine situations that could potentially promote feminine discrepancy strain would advance understanding regarding how feelings of femininity are shaped in women’s lives.

One approach to understanding the contexts in which feminine discrepancy strain commonly emerges would be to ask women open-ended questions about events in their lives that have made them feel less feminine. Additionally, expanding the strengths of the current studies, daily or weekly sampling studies could incorporate open-ended descriptions of events that made women feel more or less feminine. These approaches would develop understanding of the broad, varying, and potentially idiosyncratic experiences that create feminine gender role discrepancy strain. Moreover, returning to one of the primary contributions of the current study, such investigations are important given that the current results show FGRS and drops in felt-femininity have important consequences for women’s wellbeing.

Isolating the different contexts which promote discrepancy strain in daily and weekly life also may clarify when additional outcomes of feminine gender role strain will occur. In particular, the outcomes that emerge from feminine gender role discrepancy strain may vary based on the specific context that created feelings of gender role strain. For instance, feminine discrepancy strain may arise from the failure to embody feminine characteristics related to attractiveness, but the most prevalent outcomes of experiences of strain in this particular context are likely to be those that match the attractiveness-related domain, such as potentially increasing women’s body dissatisfaction (Harrington & Overall, 2021) and/or risk of eating disorders (Martz et al., 1995; Mussap, 2007). By contrast, discrepancy strain arising from failure to behave in nurturing ways may prompt other negative self-relevant outcomes related to negative relational evaluations, such as shame and guilt (Efthim et al., 2001).

### The Importance of Adopting a Person x Context Perspective

Prior research has primarily examined person-level and context-level gender role discrepancy strain separately, with investigations focusing on the effects of either person-level differences in gender role stress (e.g., Moore et al., 2008) or experimental manipulations of gender role discrepant contexts (e.g., Bosson et al., 2012). Both of these main effects are important. Indeed, across both studies we found that women higher in FGRS consistently experienced lower self-esteem compared to women lower in FGRS, and women who felt less feminine on a given day felt lower self-esteem compared to days they felt more feminine. Yet, our results also highlight the critical importance of taking a person x context perspective to assess the interaction between person-level and context-level gender role discrepancy strain. In particular, we demonstrated that person-level propensity for experiencing discrepancy strain (i.e., FGRS) predicted the greatest decreases in self-esteem when women encountered context-level gender role discrepant situations (i.e., lower felt-femininity).

By highlighting the importance of applying a person x context perspective when examining gender role discrepancy strain processes, the current research provides important insight into inconsistent and null effects observed in previous studies (e.g., Kosakowska-Berezecka et al., 2016; Mori et al., 1987; Munsch & Willer, 2012). Failing to account for person-level differences in propensity for feminine discrepancy strain will likely underestimate the potential impact of context-level strain or drops in felt-femininity on important outcomes. Specifically, as the current results illustrate, context-level feminine gender role discrepancy strain should have stronger effects for women higher in FGRS, and may have weaker or null effects for women lower in FGRS. Thus, mixed and null effects in tests of context-level effects (e.g., Kosakowska-Berezecka et al., 2016; Mori et al., 1987; Munsch & Willer, 2012) may emerge when such tests do not account for person-level propensity for discrepancy strain.

In the current studies we assessed FGRS to capture person-level propensity to experience feminine discrepancy strain. As feminine gender role stress measures the degree to which women find feminine gender role discrepant contexts stressful, FGRS provides a direct and concrete assessment of women’s propensity for strain in feminine gender role discrepant contexts (person-level feminine gender role discrepancy strain). Moreover, the results validate FGRS as a measure of propensity for strain in discrepant contexts by demonstrating that when women higher in FGRS experienced feminine gender role discrepancy (lower daily or weekly felt-femininity) they experienced greater strain (decreases in self-esteem) than women lower in FGRS.

Yet, the relative impact and specific outcomes of gender role discrepant situations should vary based on differences in women’s investment in specific facets of femininity (Witt & Wood, 2010; Wood & Eagly, 2009). That is, women might also differ in their propensity to experience strain within a specific discrepant context based on the extent to which the aspect of femininity they are discrepant with (e.g., attractiveness) is central to their desired identity. Thus, it also might be the case that the links between context-level discrepancy strain in specific situations and related outcomes occur more strongly for women who differ in their investment in attractiveness, communality, or other aspects of femininity (Witt & Wood, 2010; Wood & Eagly, 2009). Future research should further probe how propensity to experience strains within particular domains (e.g., attractiveness, deference, or nurturance) place women at particular risk of experiencing negative outcomes when they face context-level discrepancy strain (e.g., romantic rejection, acting assertively at work, or uncaring relationships).

By examining variation in felt-femininity across women’s daily and weekly lives, the current studies offered a direct examination of how women’s self-esteem is likely to fluctuate depending on whether women feel more or less feminine. Critically, the daily and weekly links between women’s feelings of femininity and self-esteem and were not affected by their general tendency to find challenging situations of all types stressful (i.e., not feminine-role specific; captured by MGRS). That is, although we observed a negative association between MGRS and self-esteem in Study 2, as anticipated, MGRS did not further moderate the link between women’s experiences of feminine gender role discrepancy (lower felt-femininity) and daily/weekly self-esteem. This lack of moderation supports our theorizing that women report lower self-esteem specifically when there is a match between a person-level predisposition to feminine discrepancy strain (FGRS) and context-level experience of feminine gender role discrepancy (lower felt-femininity). Taken together, our results highlight the critical importance of taking a matched person x context perspective to assess the interaction between person-level and context-level gender role discrepancy strain.

### The Importance of Examining Discrepancy Strain in Ecologically Valid Contexts

The results of the current studies provided an extension of prior research by illustrating the value of examining the outcomes of women’s experiences of feminine discrepancy strain within ecologically valid contexts that are likely to have meaningful implications for their lives. The primary approach of previous studies examining the outcomes of discrepancy strain have involved experimental manipulations designed to decrease women’s felt-femininity by providing feedback they that they are more masculine or like men (Kosakowska-Berezecka et al., 2016; Mori et al., 1987; Munsch & Willer, 2012). Yet, the inconsistent effects in prior studies suggest that this experimental approach may not effectively evoke strongly feelings of gender role discrepancy strain for women. Thus, rather than focusing on a single experience in the lab, we directly examined context-level discrepancy emerging organically in women’s daily and weekly lives; variation which is likely to stem from meaningful and impactful experiences that make women feel less feminine. Validating the strength of this approach, the results of two studies provide strong evidence that decreases in women’s feelings of femininity during daily and weekly life can undermine self-esteem, and that such outcomes of organic experiences of discrepancy strain emerge most strongly for women particularly vulnerable to feminine discrepant contexts (women higher in FGRS). Thus, the current studies illustrate both the relevance and methodological soundness of examining the outcomes of gender role discrepancy strain through naturally occurring experiences in women’s lives, and suggest that this approach may have greater power to detect feelings of gender role discrepancy strain than single, specific, and experimentally-constructed feminine discrepancy in the laboratory.

Future research examining gender role discrepancy strain for both men and women may benefit from adopting similar methods which assess naturally occurring experiences of strain, which may also allow for the assessment of contexts and outcomes not replicable in a lab setting. For instance, as feminine gender roles emphasize that women should have few sexual partners (Byers, 1996; Levant et al., 2007), women should experience feminine gender role discrepancy strain when they have sex with people they are not in a committed relationship with. Using the methods employed in the current studies, future research would be able to capture women’s experiences within this relevant context, and the outcomes of ensuing strain (e.g., such as shame and guilt; e.g., Efthim et al., 2001), which would be difficult to replicate within the constraints of a lab setting. Similarly, men’s experiences of low power within a workplace context should promote masculine discrepancy strain, particularly if their superior is a woman, and this strain could promote sexual harassment or derogation as a means of restoring men’s feelings of power and masculinity (McLaughlin et al., 2012). Assessing these kinds of reactions as they occur within relevant contexts in men’s daily and weekly lives may be more viable than assessing similar processes within a lab context, which may fail to evoke these types of expressions that emerge across ongoing relationships and could potentially place other participants at risk if enacted in the lab. Thus, future research should integrate the strengths of the current methodology and aim to capture experiences of both masculine and feminine discrepancy strain emerging within the contexts of people’s day-to-day lives.

### Limitations and Future Research Directions

Despite the strengths of the present studies, we also acknowledge some important limitations. Examining experiences as they change across real life inevitably comes along with the limitations of correlational data, preventing strong causal conclusions and leaving open the possibility of alternative explanations. Perhaps, for example, the reverse association occurs: lower self-esteem could undermine women’s self-evaluations in domains central to traditional feminine identity, such as attractiveness or nurturance, and thus decrease women’s feelings of femininity. Women more sensitive to feminine gender role discrepancy (i.e., higher in FGRS) should also find negative self-evaluations in relevant domains more challenging, and thus feel less feminine. However, we do not see this reverse association as mutually exclusive to the direction we tested. Rather, it is likely that reciprocal associations occur. Within-person reductions in felt-femininity undermine self-esteem, as we outlined, which is supported by other research showing that failure to enact desired feminine behavior is associated with decreases in self-esteem (Sanchez & Crocker, 2005; Witt & Wood, 2010). However, negative self-evaluations, especially in domains relevant to femininity, also should feedback to challenge feelings of femininity. Examining both potential causal pathways is a good direction for future research and will provide further support for the importance of the within-person associations between feelings of femininity and self-esteem (and other self-relevant outcomes).

Future research manipulating the experience of feminine discrepancy strain in ways that provide meaningful feedback may provide the strongest causal evidence. As described above, experimental manipulations providing women with feedback that they are ‘less feminine’ may not appreciably affect women’s felt-femininity, and thus future experimental designs may offer stronger tests if they administer meaningful feedback relevant to domains central to femininity and of consequence to women’s lives. For instance, women could be told that they have scored low on a test of child-care skills (discrepant with nurturance expectations), are less attractive than the average woman (discrepant with attractiveness expectations), or are perceived as unfriendly or cold by a group of people (discrepant with communality expectations). Alternatively, women could be placed in situations in which they are required to contravene feminine norms, such as a situation where they must behave assertively, take control, or argue a point. Moreover, as shown by the moderating role of FGRS in the current studies, future studies focusing on specific feminine gender role discrepant contexts should also account for women’s person-level propensity to experience gender role discrepancy strain.

Regardless of method, isolating the particular aspects and situations that lead to feminine gender role discrepancy will also advance understanding of the potential harmful outcomes women may experience when they feel less feminine. The current studies relied on a single face-valid item assessing women’s felt-femininity. This approach was informed by recent theoretical and empirical work advocating that single-item, face-valid measures are appropriate, and even preferable, in cases where the construct being measured is specific and unambiguous (see Allen et al., 2022 for review). Moreover, this approach also mirrors prior work capturing daily assessments of men’s masculinity (Overall et al., 2016) and helped minimize participant burden across repeated assessments. The item itself showed variability across participants (see Table 2), and variability across daily and weekly life, as evident in the significant within-person effect of decreases in felt-femininity on self-esteem. These results give us confidence that our assessment of felt-femininity captured important, varying, and likely idiosyncratic experiences in women’s lives that appreciably lowered feelings of femininity. Nonetheless, assessing and identifying the array of routine situations that could generate feminine gender role discrepancy strain would advance understanding regarding how femininity is shaped in women’s lives, and whether the relative impact and specific outcomes of these strain situations vary based on differences in women’s propensity for strain and investment in facets of femininity (Witt & Wood, 2010; Wood & Eagly, 2009).

We focused our studies on FGRS, and we did not assess how important femininity was to each participant’s identity. Consistent with past research, we posit that a higher endorsement of feminine ideology would associate with higher FGRS, but that FGRS (rather than feminine ideology) would account for well-being outcomes (Richmond et al., 2015). However, it is possible that women who view femininity as more central to their identity and who are higher in FGRS would show even more marked decreases in self-esteem on days or weeks when they felt less feminine (i.e., a feminine identity x FGRS x felt femininity interaction). Future research could further explore how the findings we observed intersect with the importance of one’s female identity.

Finally, the current samples involved undergraduate students. Undergraduate women represent a particularly relevant population in which to assess the links between felt-femininity and self-esteem as this developmental period is central to the development of self-esteem, particularly for women (Orth & Robins, 2014; Robins & Trzesniewski, 2005). Nonetheless, demonstrating the observed associations between women’s decreases in felt-femininity and self-esteem in younger and older populations could also provide important extensions to the current findings. For instance, replicating the results found in the current studies in samples of younger adolescents (12–18) could shed light on how decreases in felt-femininity undermine women’s self-esteem from a young age and how these outcomes affect the development of gender identities. Moreover, examining these links in older populations could identify the ways in which social pressures and expectations placed on women promote discrepancy strain, and associated decreases in self-esteem, when processes related to aging make women feel less able to embody feminine qualities, such as those related to attractiveness (e.g., Hurd, 2000).

### Practice Implications

Despite recent developments towards more egalitarian attitudes in Western society (Brewster & Padavic, 2000; Dorius & Firebaugh, 2010; Knight & Brinton, 2017), the current research suggests that young, educated Western women continue to face social expectations and pressures that place them at risk of experiencing gender role discrepancy strain. Identifying the risk feminine discrepancy strain poses to women, particularly those higher in FGRS, may offer directions for interventions targeting women’s wellbeing. Initiatives could include raising awareness of the prevalence of expectations associated with traditional feminine identity, challenging and reducing harmful proliferation of these expectations, and highlighting examples of these expectations to exemplify the common and implicit presence of the social pressures and norms women face. However, these initiatives should also account for women’s investment in feminine gender roles, as the current results highlight that the impact of feeling less feminine will be particularly challenging for women who have greater person-level propensity for experiencing feminine discrepancy strain. The results indicate that identifying women who are particularly at risk for the negative self-relevant outcomes of feeling less feminine may be most effective at protecting women’s wellbeing in the face of challenges to felt-femininity during routine life.

### Conclusion

Given the pressures women face to adhere to traditional feminine gender roles, many women may be at risk for negative self-relevant outcomes when they experience feminine gender role discrepant contexts, such as decreases in self-esteem. Moreover, the negative outcomes arising from these contextual experiences are likely to be stronger for women with greater propensity to experience feminine gender role discrepancy strain. Applying this theorizing, the current research provided the first demonstration that person-level gender role discrepancy strain (higher FGRS) and context-level gender role discrepancy strain (lower felt-femininity) combine to predict lower self-esteem in women’s daily and weekly life. Across two studies, higher FGRS (person-level discrepancy strain) and within-person decreases in daily (Study 1) and weekly (Study 2) felt-femininity (context-level discrepancy strain) predicted lower self-esteem, but higher FGRS combined with daily/weekly decreases in felt-femininity predicted the lowest self-esteem (person x context interaction). These findings illustrate the value of interventions targeting women who are particularly at risk for the negative self-relevant outcomes arising from feminine discrepancy strain, as well as the importance of identifying and counteracting the contexts that promote feminine gender role discrepancy during routine life. Future research will benefit from combining the assessment of naturally occurring decreases in feelings of femininity applied in the current studies with experimental designs to determine how person-level feminine gender role stress and experiencing gender role discrepant contexts generate negative self-relevant outcomes.

## Supplementary Information

Below is the link to the electronic supplementary material.

Supplementary file1 (DOCX 200 KB)

## Data Availability

Analysis code and materials are available in the Online Supplemental Materials (https://osf.io/gnkry/?view_only=b68776ad38db426b91a3d48e0981c046). Upon publication, data to reproduce the results will be made available on the Open Science Framework.

## Code Availability

N/A.
