# Supplementary material for: Feminine Gender Role Discrepancy Strain and Women’s Self-Esteem in Daily and Weekly Life: A Person x Context Perspective
Source: Sex Roles. 2022 Jun 13;87(1-2):35–51. doi: 10.1007/s11199-022-01305-1 (PMC9189801; doi:10.1007/s11199-022-01305-1)
Supplement: Supplementary file 1 — Supplementary file1 (DOCX 200 KB) [file 11199_2022_1305_MOESM1_ESM.docx]

Online supplement for Harrington, A.G., Overall, N.C., & Maxwell, J.A. (2022). Feminine gender role discrepancy strain and women’s self-esteem in daily and weekly life: A person x context perspective. *Sex Roles*. Auguste Glenn Harrington, University of Auckland. Email: [a.harrington@auckland.ac.nz](mailto:a.harrington@auckland.ac.nz)

**Contents**

1. Feminine Gender Role Stress Items (Studies 1 and 2) 2

2. Masculine Gender Role Stress Items (Study 2) 3

3. SPSS Syntax for Analyses Presented in Table 2 (Study 1) 5

4. SPSS Syntax for Analyses Presented in Table 2 (Study 2) 6

5. Examining Sub-Samples Gathered Prior versus Post COVID-19 Pandemic (Study 2) 7

6. References 11


**1. Feminine Gender Role Stress Items (Studies 1 and 2)**

Our assessment of FGRS included 24 of the original 39 items to maximize attentive responding given the data collection paradigm and align with abbreviated measures now used to assess MGRS. Our primary approach was to select 5 items from each of the 5 subscales. Items removed were those that (1) were very similar to other high-loading items from the original scale development (Gillespie & Eisler, 1992), (2) involved situations that are not widely generalizable (e.g., Being unusually tall), or (3) we judged were likely to be very stressful for everyone and thus may not as sensitively assess level of threat to women’s feminine identity (e.g., Hearing that a dangerous criminal has escaped nearby).

| **Sub Scale of the Feminine Gender Role Stress Scale** (Gillespie & Eisler, 1992) | **FGRS Items in Studies 1 and 2** | **FGRS Items Removed from Each Factor** |
| --- | --- | --- |
| **Fear of Unemotional Relationships** | Being considered promiscuous  Having others believe that you are emotionally cold  Being pressured for sex when seeking affection from your intimate partner  Not being able to meet family members' emotional needs  Your intimate partner will not discuss your relationship problems | Feeling pressured to engage in sexual activity  Having to deal with unwanted sexual advances  Being taken for granted in a sexual relationship  Having multiple sex partners  Having an intimate relationship without any romance |
| **Fear of Physical Unattractiveness** | Being perceived by others as overweight  Finding out that you gained 10 pounds  Turning middle-aged and being single  Feeling less attractive than you once were  Wearing a bathing suit in public  Being unable to change your appearance to please someone | Being heavier than your mate  Being unusually tall |
| **Fear of Victimization** | Hearing a strange noise while you are home alone  Having your car break down on the road  Feeling that you are being followed by someone  Receiving an obscene phone call | Hearing that a dangerous criminal has escaped nearby  Having to move to a new city or town alone  Bargaining with a salesperson when buying a car |
| **Fear of Behaving Assertively** | Talking with someone who is angry with you  Supervising older and more experienced employees at work  Having to "sell" yourself at a job interview  Making sure you are not taken advantage of when buying a house or car  Bargaining with a salesperson when buying a car | Negotiating the price of car repairs  Trying to be a good parent and excel at work |
| **Fear of Not Being Nurturant** | A very close friend stops speaking to you  Losing custody of your children after divorce  Your child is disliked by his or her peers  Returning to work soon after your child is born | Your mate is unemployed and cannot find a job  Having a weak or incompetent spouse  Having someone else raise your children  Trying to get your spouse to take responsibility for childcare |

**2. Masculine Gender Role Stress Items (Study 2)**

In Study 2, participants completed a more detailed assessment of MGRS than the abbreviated MGRS scale (Swartout et al., 2015) which consisted of 30 of the original 40 items (Eisler & Skidmore, 1987). Participants rated each item according to how stressful they would find each situation to be if they were in that situation (1 = *not at all stressful*, 7 = *extremely stressful*). Our primary approach was to remove 2 items from each of the 5 factors. Items removed were those that (1) were very similar to other higher-loading items from the original scale development (Eisler & Skidmore, 1987) or (2) referred to children and thus implied parental status, which may not be relevant to some or many of our participants.

| **Sub Scale of the Masculine Gender Role Stress Scale** (Eisler & Skidmore, 1987) | **MGRS Items in Study 2** | **MGRS Items Removed from each Factor** |
| --- | --- | --- |
| **Physical Inadequacy** | Not being able to find a sexual partner  Having your lover say that she/he is not satisfied  Being perceived by someone as "gay"  Losing in a sports competition  Being perceived as having feminine traits  Appearing less athletic than a friend  Knowing you cannot hold your liquor as well as others | Feeling that you are not in good physical condition  Being compared unfavorably to men |
| **Emotional Inexpressiveness** | Telling someone that you feel hurt by what she/he said  Admitting that you are afraid of something  Talking with a woman who is crying  Comforting a male friend who is upset | Telling your spouse that you love her/him  Having your children see you cry  Having a man put his arm around your shoulder |
| **Subordination to Women** | Being outperformed at work by a woman  Having a female boss  Letting a woman take control of the situation  Being outperformed in a game by a woman  Being with a romantic partner who is much taller than you  Needing your partner to work to help support the family  Admitting to your friends that you do housework  Being married to someone who makes more money than you | Being with a woman who is more successful than you |
| **Intellectual Inferiority** | Having to ask for directions when you are lost  Talking with a ‘feminist’  Having people say that you are indecisive  Having others say that you are too emotional  Working with people who are brighter than yourself | Working with people who seem more ambitious than you  Staying home during the day with a sick child |
| **Performance Failure** | Not making enough money  Finding you lack the occupational skills to succeed  Being unable to perform sexually  Being too tired for sex when your lover initiates it  Being unable to become sexual aroused when you want  Getting passed over for a promotion | Being unemployed  Getting fired from your job |

**4. SPSS Syntax for Analyses Presented in Table 2 (Study 1)**

In Study 1 we used SPSS 26 to estimate the model following the syntax and procedures outlined by Bolger and Laurenceau (2013). Below we present the SPSS syntax used to test whether women’s greater feminine gender role stress and decreases daily feelings of femininity combine to predict lower daily feelings of self-esteem. Lowercase is a variable, and uppercase is required SPSS syntax.

MIXED dailyselfesteem WITH FGRS_c dailyfemininty_pc
/FIXED= FGRS_c dailyfemininty_pc FGRS_c*dailyfemininty_pc
/PRINT= SOLUTION TESTCOV COVB
/RANDOM= intercept | SUBJECT(particpantno) COVTYPE(UN)
/REPEATED= responseday | SUBJECT(participantno) COVTYPE(AR1).

This syntax specifies a multi-level model for analyzing repeated assessments nested within each participant (participantno). The REPEATED statement treats each daily assessment as repeated measures within each participant to account for the nonindependence across the daily assessments for each participant. The AR1 term specifies an autoregressive error structure. Essentially, the model accounts for the within-person associations across each daily report of the dependent variable (in this case self-esteem) so that any predictor of the dependent variable does not over-estimate effects that arise from correlated repeated assessments from the same person.

The MIXED line specifies the structure of the multilevel model. The variable *dailyselfesteem* represents the daily assessments of self-esteem participants experienced that day and is the dependent variable in this model. The remaining variables on the first line (following WITH) are those included in the model to specify the fixed effects of predictors and moderators: *FGRS_c* is participants’ feminine gender role stress when entering the study (grand-mean centered); *dailyfemininty_pc* is participants’ reported feelings of femininity that day (person-mean centered).

The FIXED line estimates the effects of each variable and interaction term predicting self-esteem presented in Table 2. *FGRS_c* tests whether women higher in feminine gender role stress have generally lower daily feelings of self-esteem. *Dailyfeminininty_pc* models the effects of feelings of femininity, which was person-centered such that the coefficient models whether variations in daily feelings of femininity from person’s typical levels of femininity predict decreases in self-esteem that day. The *FGRS_c*dailyfemininity_pc* interaction tests if feminine gender role stress and within-person changes in daily feelings of femininty combine to predict self-esteem. The RANDOM line specifies that the intercept (average *dailyselfesteem*) is modelled as a random effect, and thus models how much daily self-esteem varies across participants.

Additional analyses testing whether our effect was independent of masculine gender role stress were run with an identical analytic strategy to that presented above except that MGRS_c and the *MGRS_*c**dailyfemininity* interaction were added.

**5. SPSS Syntax for Analyses Presented in Table 2 (Study 2)**

In Study 2 we once again used SPSS 26 to estimate the model following the syntax and procedures outlined by Bolger and Laurenceau (2013). Below we present the SPSS syntax used to test whether greater feminine gender role stress and decreases in weekly feelings of femininity combine to predict lower weekly feelings of self-esteem. Lowercase is a variable, and uppercase is required SPSS syntax.

MIXED weeklyselfesteem WITH FGRS_c weeklyfemininity_pc
/FIXED= FGRS_c weeklyfemininity_pc FGRS_c*weeklyfemininity_pc*
/PRINT= SOLUTION TESTCOV COVB
/RANDOM= intercept | SUBJECT(particpantno) COVTYPE(UN)
/REPEATED= responseweek | SUBJECT(participantno) COVTYPE(AR1).

This syntax specifies a multi-level model for analyzing repeated assessments nested within each participant (participantno). The REPEATED statement treats each weekly assessment as repeated measures within each participant to account for the nonindependence across the weekly assessments for each participant. The AR1 term specifies an autoregressive error structure. Essentially, the model accounts for the within-person associations across each weekly report of the dependent variable (in this case self-esteem) so that any predictor of the dependent variable does not over-estimate effects that arise from correlated repeated assessments from the same person.

The MIXED line specifies the structure of the multilevel model. The variable *weeklyselfesteem* represents the weekly assessments of self-esteem participants experienced that week and is the dependent variable in this model. The remaining variables on the first line (following WITH) are those included in the model to specify the fixed effects of predictors and moderators: *FGRS_c* is participants’ feminine gender role stress when entering the study (grand-mean centered); *weeklyfemininity_pc* is participants’ reported feelings of femininity that week (person-mean centered).

The FIXED line estimates the effects of each variable and interaction term predicting self-esteem presented in Table 2. *FGRS_c* tests whether women higher in FGRS have generally lower self-esteem. *Weeklyfemininity_pc* models the effects of feelings of femininity that week, which was person-centered such that the coefficient models whether variations in weekly feelings of femininity from person’s typical levels of femininity predict decreases in self-esteem that week. The *FGRS_c***weeklyfemininity_pc* interaction tests if feminine gender role stress and within-person changes in weekly feelings of femininty combine to predict self-esteem. The RANDOM line specifies that the intercept (average levels of *weeklyselfesteem*) is modelled as a random effect, and thus how much weekly self-esteem varies across participants.

Additional analyses testing whether our effect was independent of masculine gender role stress were run with an identical analytic strategy to that presented above except that *MGRS_c* and the *MGRS_c*weeklyfemininity*interaction were added.

**6. Examining Sub-Samples Gathered Prior versus Post COVID-19 Pandemic (Study 2)**

As in Study 1, we recruited a large sample by running Study 2 across three academic semesters (N = 165). Two semesters occurred in 2019 prior to the emergence of the COVID-19 pandemic, and the third occurred after the COVID-19 had initially been eliminated in the community in 2020. However, 80 participants sampled in 2020 experienced a short lockdown (18 days) during the data collection period. We included all data for transparency and to maximize statistical power, and because we did not have firm a priori expectations that the post-COVID semester would have weaker (minimize femininity threat) or stronger (amplify femininity threat) effects. Here we provide the results of analyses examining whether the main and interaction effects of felt-femininity and FGRS on weekly self-esteem differed between responses collected in 2019 and those collected in 2020. To do this, we added the main and interaction effects of year sampled (*0 =* 2019, *1 =* 2020) to the main analyses reported in Table 2. These results are shown in SM Table 1 on page 8 of this document. The main and interaction effects of felt-femininity and FGRS did not significantly differ across data collected in 2019 versus 2020. Nonetheless, as the Feminine Gender Role Stress X Weekly Feelings of Femininity X Year 3-way interaction was *p* = .062, we tested the main and interaction effects separately for the 2019 semesters and 2020 semesters. The results are shown in SM Table 2 and SM Figure 1 (on pages 9 and 10 of this document). The interaction effect was weaker (and non-significant) in the 2020 sample. However, as is clearly visible in SM Figure 1, this was because the main effect of FGRS on lower self-esteem was stronger in the sample that may have faced more stress, and this main effect was not reduced when felt-femininity was high. Thus, the results continued to support that within-person reductions in felt-femininity was associated with concomitant decreases in lower self-esteem, and greater FGRS predicted lower self-esteem even when felt-femininity was high.

Study 1 was collected from students in a third-year class between July 2019 and March 2020, whereas Study 2 was collected from a sample of students in second year classes who elected to participate in the study for course credit (amongst a range of other study options) between March 2019 and July 202. Taking into account the typical course progression of students, it is possible that there is a maximum of 66 participants who may have who may have participated in one wave of Study 2 (2019) as well as one wave of Study 1 (2020). We cannot identify these potential overlapping participants due to their responses being anonymous. However, the moderations presented in this section also captures the subset of the samples who might have overlapped, which were all coded as pre-COVID-19. Thus, the lack of moderation by COVID-19 timing suggests our results are not being driven by this potential overlap.

**SM Table 1**

*Women’s Feminine Gender Role Stress and Weekly Feelings of Femininity Predicting Weekly Self-Esteem Controlling for Year Sampled (Study 2)*

| **Variables** | *B* | *95% CI* | |  |  |  |
| --- | --- | --- | --- | --- | --- | --- |
|  |  | Lower | Upper | *t* | *p* | *r* |
| Feminine Gender Role Stress | -.561 | -.918 | -.203 | -3.095 | .002 | .237 |
| Weekly Feelings of Femininity | .217 | .134 | .299 | 5.143 | .000 | .168 |
| Year | .159 | -.213 | .530 | .845 | .400 | .066 |
| Feminine Gender Role Stress X  Weekly Feelings of Femininity | .191 | .070 | .312 | 3.107 | .002 | .102 |
| Feminine Gender Role Stress X Year | -.058 | -.605 | .490 | -.208 | .836 | .016 |
| Weekly Feelings of Femininity X Year | -.002 | -.118 | .115 | -.030 | .976 | .001 |
| Feminine Gender Role Stress X Weekly Feelings of Femininity X Year | -.173 | -.355 | .009 | -1.869 | .062 | .062 |

*Note*. *CI* = Confidence Interval. Year coded as: *0 =* 2019, *1 =* 2020. Effect sizes (*r*) were computed using Rosenthal and Rosnow’s (2008) formula: *r* = √(*t* 2 / *t* 2 + *df*). In these multilevel models, the Satterthwaite approximation is applied to provide specific degrees of freedom for each effect representing the weighted average of the between and within-person degrees of freedom, which were used to calculate the effect sizes.

**SM Table 2***Women’s Feminine Gender Role Stress and Weekly Feelings of Femininity Predicting Weekly Self-Esteem Split by Year Sampled: Study 2*

| **Variables** | *B* | *95% CI* | |  |  |  |
| --- | --- | --- | --- | --- | --- | --- |
|  |  | Lower | Upper | *t* | *p* | *r* |
| **2019** |  |  |  |  |  |  |
| Feminine Gender Role Stress | -.560 | -.933 | -.187 | -2.988 | .004 | .312 |
| Weekly Feelings of Femininity | .210 | .127 | .293 | 4.991 | .000 | .227 |
| Feminine Gender Role Stress X Weekly Feelings of Femininity | .193 | .072 | .314 | 3.137 | .002 | .144 |
| **2020** |  |  |  |  |  |  |
| Feminine Gender Role Stress | -.616 | -1.018 | -.214 | -3.051 | .003 | .326 |
| Weekly Feelings of Femininity | .221 | .139 | .303 | 5.310 | .000 | .243 |
| Feminine Gender Role Stress X Weekly Feelings of Femininity | .013 | -.123 | .148 | .185 | .853 | .009 |

*Note*. *CI* = Confidence Interval. Year coded as: *0 =* 2019, *1 =* 2020. Effect sizes (*r*) were computed using Rosenthal and Rosnow’s (2008) formula: *r* = √(*t* 2 / *t* 2 + *df*). In these multilevel models, the Satterthwaite approximation is applied to provide specific degrees of freedom for each effect representing the weighted average of the between and within-person degrees of freedom, which were used to calculate the effect sizes.

**Figure SM1**

*The Person X Context Interaction Between FGRS and Women’s Weekly Feelings of Femininity Predicting Self-Esteem Split by Year Sampled: Study 2*


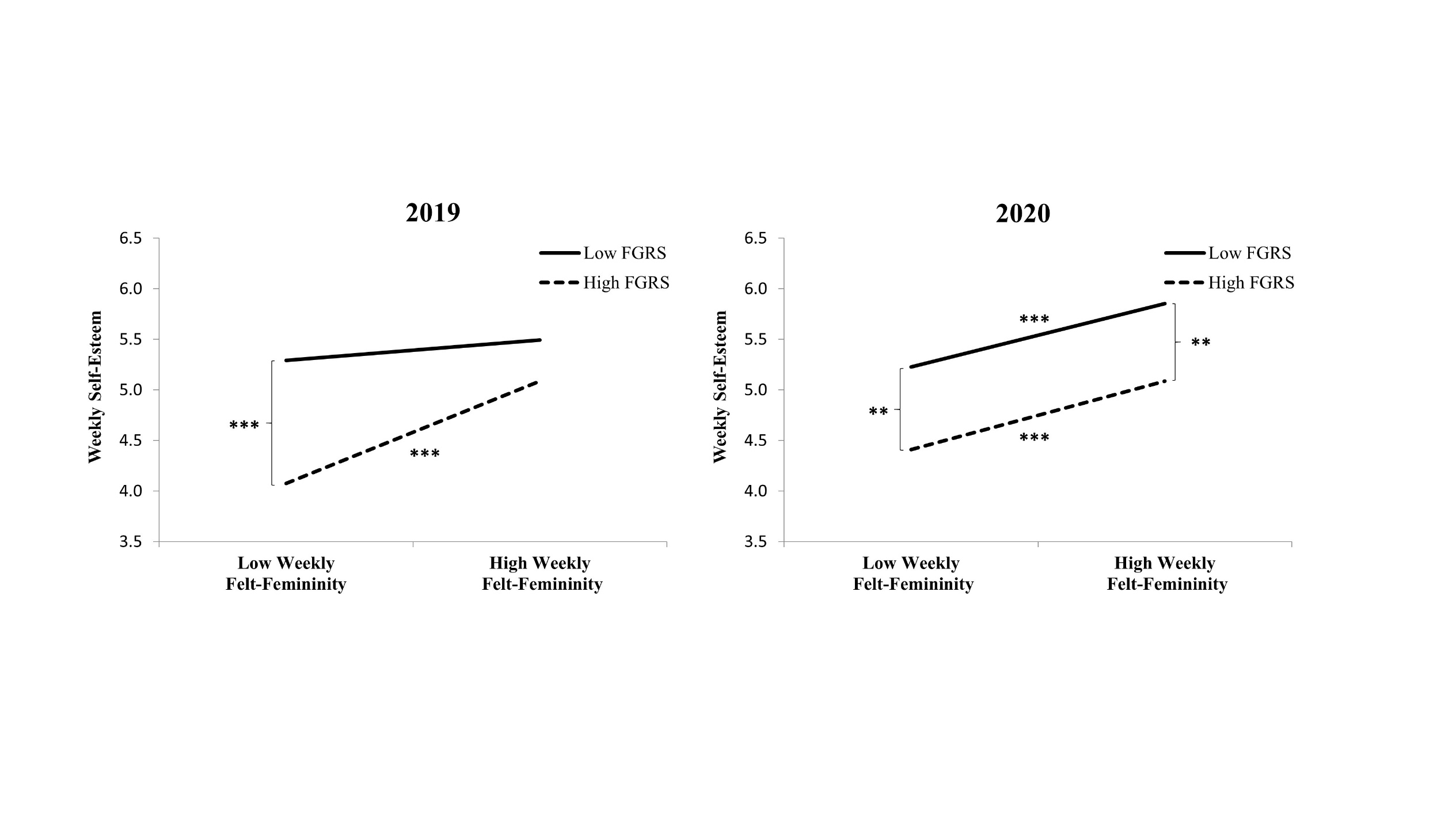


*Note.* FGRS = Feminine Gender Role Stress. High and low values represent 1 SD above and below the mean. ***slopes and simple effects significant at *p* < .001*. *** Simple effects significant at *p* < .01*.*

**References**

Bolger, N., & Laurenceau, J. P. (2013). *Intensive longitudinal methods: An introduction to diary and experience sampling research*. Guilford Press.

Eisler, R. M., & Skidmore, J. R. (1987). Masculine gender role stress: Scale development and component factors in the appraisal of stressful situations. *Behavior Modification*, *11*(2), 123-136.

Gillespie, B. L., & Eisler, R. M. (1992). Development of the feminine gender role stress scale: A cognitive-behavioral measure of stress, appraisal, and coping for women. *Behavior Modification*, *16*(3), 426-438.

Rosnow, R. L., & Rosenthal, R. (2008). *Assessing the effect size of outcome research.* Oxford University Press.

Swartout, K. M., Parrott, D. J., Cohn, A. M., Hagman, B. T., & Gallagher, K. E. (2015). Development of the abbreviated masculine gender role stress scale. *Psychological Assessment*, *27*(2), 489.
